# Facile synthesis of aminated indole-based porous organic polymer for highly selective capture of CO_2_ by the coefficient effect of π–π-stacking and hydrogen bonding†

**DOI:** 10.1039/c9ra01532a

**Published:** 2019-04-16

**Authors:** Qiang He, Yi Xu, Xiaoqiang Yang

**Affiliations:** School of Aviation Engineering Institute, Civil Aviation Flight University of China Guanghan 618307 People's Republic of China heqiangxy@126.com

## Abstract

A new aromatic aminated indole-based porous organic polymer, PIN-NH_2_, has been successfully constructed, and it was demonstrated that the coefficient effect endows this porous material with outstanding CO_2_ absorption capacity (27.7 wt%, 1.0 bar, 273 K) and high CO_2_/N_2_ (137 at 273 K and 1 bar) and CO_2_/CH_4_ (34 at 273 K and 1 bar) selectivity.

## Abstract 2

It was demonstrated that the coefficient effect endows POP PIN-NH_2_ with outstanding CO_2_ absorption capacity and high selectivity.

## Graphical abstract

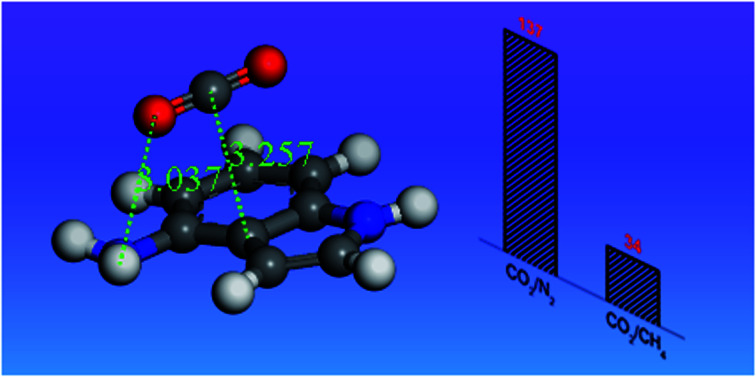

Today, one of the most serious environmental problems is climate change, such as global warming and sea-level rises, which are caused by increased concentrations of carbon dioxide (CO_2_) in the atmosphere.^1–3^ As we all know, CO_2_ mainly arises from fossil-fuel combustion in power plants, and the flue gas is always mixed with other gases including nitrogen (N_2_), methane (CH_4_) and so on. Therefore, it is necessary to design materials for selectively separating and adsorbing CO_2_ from these industrial and energy-related sources to improve the environmental problems.^4–6^ Aqueous amine solutions are the most common adsorbents for CO_2_ separation and capture,^7^ however, not only do these adsorbents degrade over time and are corrosive, toxic, and volatile, but also the regeneration process is highly energy demanding for these systems due to the chemical capture mechanism. As alternatives, porous organic polymers (POPs)^8–10^ relying on physical adsorption have become the research focus due to their low density, large specific surface area, good thermal stability, and narrow pore size distribution, but the low uptake capacity, and especially, the poor selectivity are two urgent issues that need to be addressed that seriously restrict the commercialization of POP adsorbents.^11^ Hence, in the past few years, many methods have been developed to improve the POP performance including increasing the surface area and adjusting the pore size.^12,13^

Recently, based on the rapid development of supramolecular interactions^14,15^ and the unique advantage of POP materials, *i.e.*, the structure designability, researchers found that introducing special active sites into the framework, such as heteroatoms and diverse organic groups, is a simple and effective way to ameliorate the adsorption performance by the formation of some special non-covalent interactions and various functional groups have been explored.^16–18^ Recently, Chang *et al.*^19^ have designed and prepared an novel aerogel (PINAA) that contains both amide and indole groups and they demonstrated that the CO_2_ can be rapidly adsorbed on the heteroaromatic ring of indole because of its relatively large binding area *via* strong π–π-stacking interactions, and then, the desorbed CO_2_ molecule can be captured by an adjacent amide group because of “electrostatic in-plane” interaction. This synergistic effect of electrostatic in-plane and dispersive π–π-stacking interactions of amide and indole with CO_2_ endows the resulting aerogel enhanced CO_2_ adsorption capacity and CO_2_/CH_4_ and CO_2_/N_2_ selectivity. Inspired by this fascinating study, we hypothesized that when the indole group is aminated, the CO_2_ can be rapidly adsorbed on the heteroaromatic ring of indole because of its relatively large binding area *via* strong π–π-stacking interaction (Fig. 1a), after that, the hydrogen bonding interactions between the O of the CO_2_ and –NH of the aniline group would make the CO_2_ to further form a stable conformation with the aminated indole system (Fig. 1b), as a result, the coefficient effect of π–π-stacking interactions and hydrogen bonding interactions would ensure the high CO_2_ adsorption capacity and further enhanced CO_2_/CH_4_ and CO_2_/N_2_ selectivity.

**Fig. 1:**
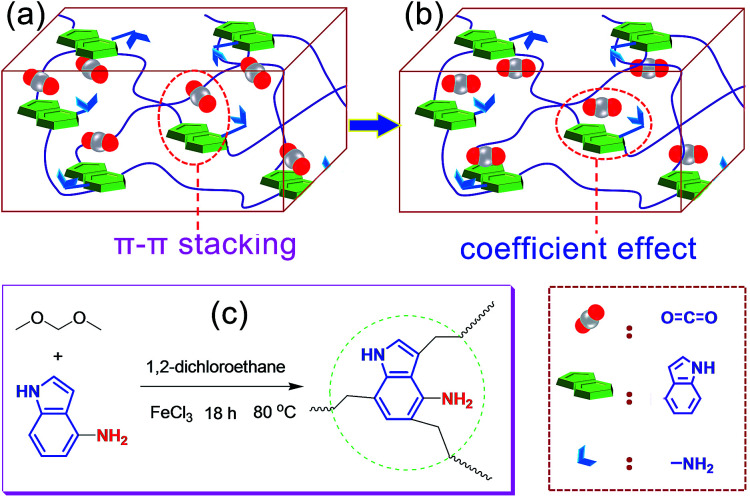
Schematic representation showing the heteroaromatic ring of indole adsorbing CO_2_*via* π–π-stacking interactions (a) and the CO_2_ molecule is further stabled *via* the coefficient effect of π–π-stacking interactions and hydrogen bonding interactions (b). (c) Synthetic route of PIN-NH_2_ aerogel.

To verify our suppose, in this work, we tactfully designed and fabricated an aminated indole-based aerogel PIN-NH_2_*via* Friedel–Crafts alkylation (Fig. 1c), and its CO_2_ adsorption capacity and CO_2_/CH_4_ and CO_2_/N_2_ selectivity were immediately investigated. The successful preparation of PIN-NH_2_ was confirmed by Fourier transform infrared spectroscopy (FT-IR) and ^13^C solid state cross-polarization magic-angle-spinning nuclear magnetic resonance (^13^C CP/MAS NMR) spectrometer, and the results are in good agreement with the proposed structures (Fig. S1 and S2, ESI†). In the ^13^C CP/MAS NMR spectrum of PIN-NH_2_, the peaks at 169–103 ppm are ascribed to the indole group carbons, and the signals located at 35–40 ppm are assigned to the methylene carbons (Fig. S1, ESI†). For FT-IR spectrum (Fig. S2, ESI†), the peak at 3438 cm^−1^ is attributed to the stretching vibrations of N–H in amine unit and indole amine. The peaks at 2999 cm^−1^ and 2927 cm^−1^ are assigned to the stretching vibration of –CH_2_– in the polymer network and the peaks at 1630 cm^−1^ and 1480 cm^−1^ are ascribed to the vibrations of the aromatic ring skeleton.

The porosity of PIN-NH_2_ was quantified by scanning electron microscopy (SEM), high-resolution transmission electron microscopy (TEM) and N_2_ adsorption–desorption isotherms at 77 K. As shown in Fig. 2a, the SEM image displays that the PIN-NH_2_ consists of aggregated particles with sub-micrometer sizes. And the microporous characteristic can be observed clearly from the TEM image as shown in Fig. 2b, the presence of porous structure provides the essential condition for CO_2_ capture and separation. As shown in Fig. 2c, at a low pressure (0–0.1 bar), there is a rapid raise in the N_2_ adsorption–desorption isotherm, indicating its microporous nature, and the increase in the N_2_ sorption at a relatively high pressure (∼0.9 bar) shows the presence of meso- and macrostructures of the PIN-NH_2_. The specific surface area calculated in the relative pressure (*P*/*P*_0_) range from 0.01 to 0.1 shows that the Brunauer–Emmett–Teller (BET) specific surface area of PIN-NH_2_ is up to 480 m^2^ g^−1^. Additionally, the pore-size distribution (PSD)^20^ calculation result was shown in Fig. 2d and S3 ESI,† which indicating the pore diameter is about 14 Å and further confirming the microporous feature of the PIN-NH_2_. To gain further insight into the microstructural information, the powder wide-angle X-ray diffraction (PXRD) was further performed on the PIN-NH_2_ polymer. As shown in Fig. S4, ESI,† only a broad peak at 17.8° 2*θ* in the PXRD pattern is present, which clearly suggests that the polymer is mainly amorphous in nature. Additional, the thermogravimetric analysis (TGA) show that the microporous material is stable up to 370 °C indicating its potential in post combustion processes operated at high temperatures (Fig. S5, ESI†).

**Fig. 2:**
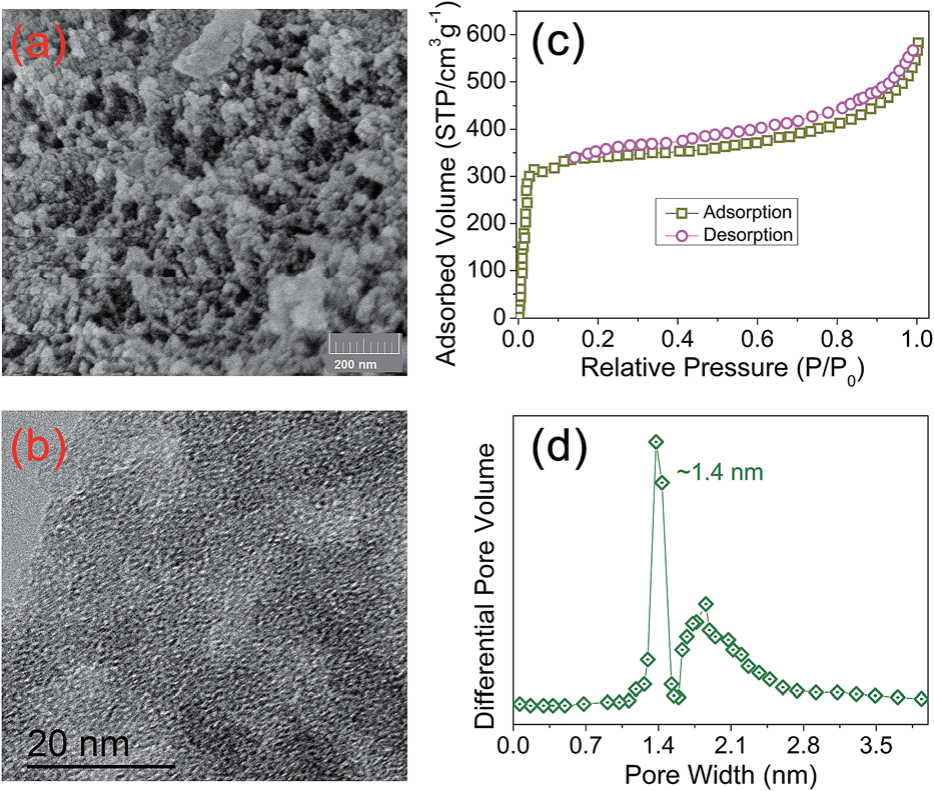
The microstructures of PIN-NH_2_ framework. (a) SEM, (b) TEM, (c) the nitrogen adsorption–desorption isotherms and (d) the pore size distribution of PIN-NH_2_ framework.

Owing to the artful structure design and particular preparation method, there is a reserved aniline group on the side of the indole group in the PIN-NH_2_ network. It was expected that after the rapidly capture of the CO_2_ molecule *via* the π–π-stacking interactions, the next aniline group would assist to further stabilize the CO_2_ molecule *via* hydrogen bonding interactions, in other words, the coefficient effect of π–π-stacking interactions and hydrogen bonding interactions would make this porous organic polymer more efficiently attract CO_2_ molecules, which inspires us to investigate the gas uptake capacity. Physisorption isotherms for CO_2_ (at 273 K) measured with a pressure more than 1.0 bar indicated that the resulting PIN-NH_2_ network exhibited a high carbon dioxide uptake of 27.7 wt% at 1.0 bar, as shown in Fig. 3a. Comparing with most of reported porous materials such as metal–organic frameworks,^21^ activated carbons,^22^ and microporous organic polymers,^23,24^ the porous organic polymer PIN-NH_2_ shows an enhanced CO_2_ uptake (Table S1, ESI†). The calculation of isosteric heat of adsorption of PIN-NH_2_ shows that the heat of adsorption is 35.7 kJ mol^−1^ (Fig. S6, ESI†), which is higher than that of the reported azo-linked polymers (27.9–29.6 kJ mol^−1^),^25^ the acid-functionalized porous polymers (32.6 kJ mol^−1^).^26,27^ and the indole-based porous polymers.^28,29^ The high value of the heat of adsorption indicated the strong physisorption effect owing to the coefficient effect of π–π-stacking interactions and hydrogen bonding interactions.

**Fig. 3:**
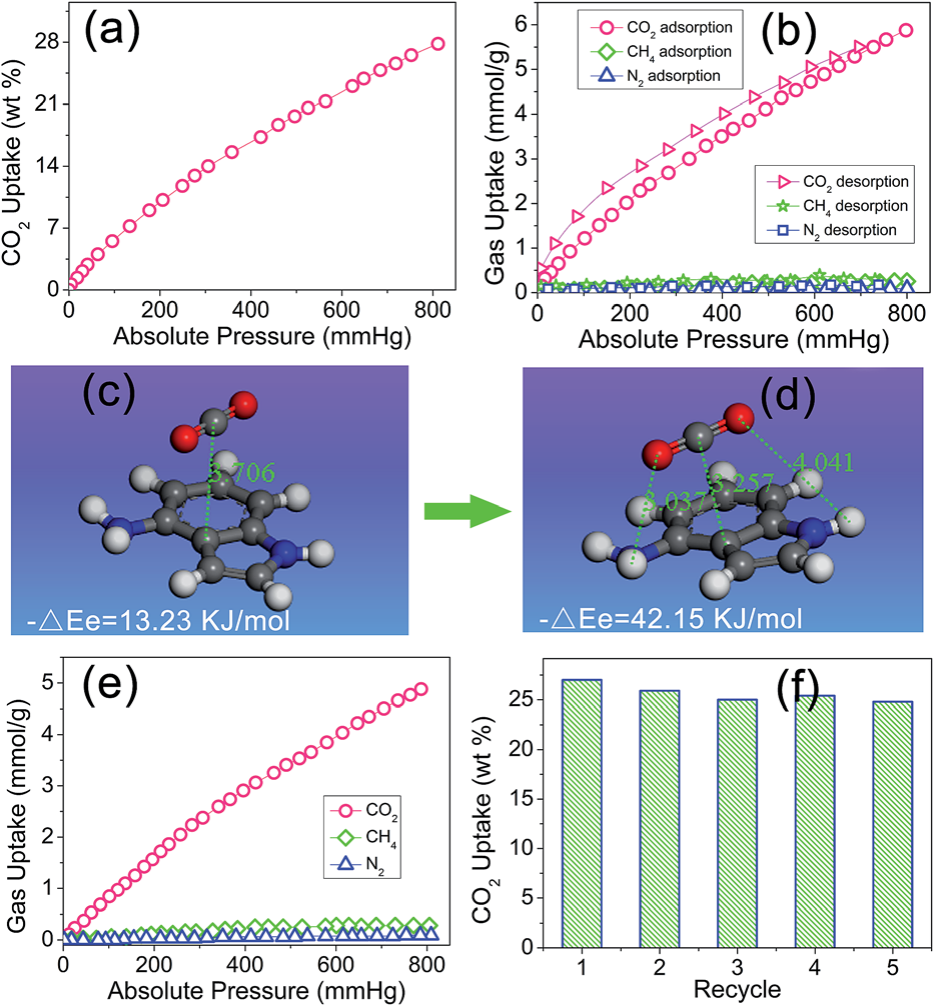
Gas adsorption isotherms of PIN-NH_2_ for CO_2_ at 273 K (a), adsorption and desorption isotherms of PIN-NH_2_ for different gases at 273 K (b), a CO_2_ molecule is adsorbed on the heteroaromatic ring of indole *via* π–π-stacking interaction (c) and the CO_2_ molecule is further stabled *via* the coefficient effect of π–π-stacking and hydrogen bonding interactions (d), adsorption isotherms of PIN-NH_2_ for different gases with 3% RH of water at 273 K (e), reversibility of the PIN-NH_2_ polymer in CO_2_ capture measured by TGA at 273 K (f).

The application in CO_2_ separation and adsorption field of the traditional POPs is limited in a great degree by the poor gas selectivity as the flue gas and natural gas are both mixed gas. Here, we believed that the CO_2_ can be easily attracted by the heteroaromatic ring *via* the π–π-stacking interactions and then stabled with the assist of hydrogen bonding interactions, which leading an enhanced gas selectivity. Therefore, we urgently evaluated the selective gas uptake of the PIN-NH_2_ network for small gases (CO_2_/CH_4_, CO_2_/N_2_). In the calculation, the ratio of CO_2_/N_2_ is 15/85 and the ratio of CO_2_/CH_4_ is 5/95, which is the typical composition of flue gas and natural gas, respectively, the test results were shown in Fig. 3b. It can be found there is a rapid increase for the CO_2_ uptake while there is a negligible increase for the CH_4_ and N_2_ uptake with the increase of the pressure, which maybe due to the unique local dipole–π interactions between the porous organic framework PIN-NH_2_ and CO_2_ molecule. The test results shows that the CO_2_ uptake of PIN-NH_2_ is up to 5.92 mmol g^−1^ at a pressure of 1.0 bar and a temperature of 273 K while the CH_4_ and N_2_ uptake of PIN-NH_2_ is only 0.18 and 0.04 mmol g^−1^, respectively. The estimated ideal CO_2_/CH_4_ and CO_2_/N_2_ adsorption selectivities are up to 34 and 137, respectively. Additionally, the selectivities of PIN-NH_2_ toward CO_2_ over CH_4_ and N_2_ at 291 and 303 K were also investigated, respectively, and the results indicated that the resulting polymer PIN-NH_2_ still exhibited good selectivity at higher temperatures (Fig. S7 and S8, ESI†).

The high gas selectivities of this microporous framework may attribute to the strong affinity for CO_2_ compared with N_2_ and CH_4_ arising from the coefficient effect of π–π-stacking interactions and hydrogen bonding interactions between the sorbent and CO_2_ guest molecule, to further attest the above surmise, we used density functional theory (DFT)^30^ at the M06-2X level with the aug-cc-pVDZ basis set to investigate the interaction of aminated indole system with CO_2_ and the details of the calculation is shown in the ESI.†Fig. 3c and d shows the snapshot for CO_2_ capture by a model compound. The calculation result shows that owing to the electron-rich and large binding area, the CO_2_ was very easily attracted by the indole plane at a distance of 3.706 Å, and the computational binding energy was 13.23 kJ mol^−1^ (Fig. 3c). Soon, the balance structure was changed, the CO_2_ molecular was moved towards the amino group till the distance between the amino group and CO_2_ molecular was 3.037 Å, indicating a hydrogen bonding interaction was formed in this system. As a result, the distance between the indole plane and CO_2_ molecular decreased to 3.257 Å from 3.706 Å, and the computational binding energy increased to 42.15 kJ mol^−1^, which meaning a more steady system was formed (Fig. 3d). In the sense of computational chemistry, the expected strong coefficient effect of π–π-stacking interactions and hydrogen bonding interactions would favor the uptake of CO_2_ of the PIN-NH_2_ network. Additional, The DFT result also indicated that the interaction energy between CO_2_ and the imine group of indole is relatively weak with a correlation distance at 4.041 Å.

As we all know that the CO_2_ adsorption property will be affected in a great degree for porous polymers in the presence of water.^31^ In real industrial applications, the flue gas from a power plant is a mixture of CO_2_, water vapor, and others. As a result, it has very important practical significance to study the CO_2_ capture performance under humid condition. Here, the CO_2_ capture property of PIN-NH_2_ was studied at a relative humidity of 3% RH, as shown in Fig. 3e, the CO_2_ adsorption capacity of PIN-NH_2_ decreased from 5.92 to 4.88 mmol g^−1^ (1.0 bar, 273 K), however, the uptake of CH_4_ and N_2_ does not affected by the water. These results indicate that adsorption of water diminishes the CO_2_ capture. Although the selectivity (CO_2_/N_2_ = 104, CO_2_/CH_4_ = 21) is decreased under humid condition, PIN-NH_2_, to the best of our knowledge, still has very good CO_2_ selectivity over other CO_2_ capture materials in similar conditions.^32^ Moreover, the CO_2_ adsorption process is fully reversible (Fig. 3f). Herein, the new aminated indole-based aromatic porous organic polymer PIN-NH_2_ synthesized from easily available starting materials demonstrated not only remarkable CO_2_ capture capacity, but also prominent CO_2_/N_2_ and CO_2_/CH_4_ selectivities. Further, the dynamic breakthrough separation experiments of gas mixture at 298 K using a fixed-bed column packed with PIN-NH_2_ was carried out to evaluate the performances of PIN-NH_2_ aerogel in an actual adsorption-based separation process. The details of the experiment process were described in ESI.† As shown in Fig. S9 and S10, ESI,† the CH_4_ and N_2_ penetrated through the bed firstly with a retention time for only 6.5 and 3.4 min, respectively, while PIN-NH_2_ column can retain CO_2_ until above 23 min, which means the high CO_2_ adsorption capacity and selectivity of the PIN-NH_2_ adsorbent in actual application.

## Conclusions

In this work, we have designed and synthesized a novel aromatic aminated indole-based porous organic polymer PIN-NH_2_*via* Friedel–Crafts alkylation of 4-aminoindole with formaldehyde dimethyl acetal. FTIR and ^13^C CP/MAS NMR characterizations were performed to study the structural information and confirmed the successful formation of the resulting porous organic polymer PIN-NH_2_. The nitrogen adsorption–desorption test shows that the Brunauer–Emmett–Teller (BET) specific surface area of PIN-NH_2_ is up to 480 m^2^ g^−1^ and the thermal analysis indicates that the PIN-NH_2_ possesses good thermal stability. More interestingly, we proved that the CO_2_ adsorption capacity (27.7 wt%, 1.0 bar, 273 K) and selectivities (CO_2_/N_2_ = 137, CO_2_/CH_4_ = 34) could be significantly improved may owing to the presence of coefficient effect of π–π-stacking interactions and hydrogen bonding interactions between the sorbent and CO_2_ guest molecule, making it a promising material for potential application in gas separation. In addition, upon exposure to moisture (RH = 3%), CO_2_ capture of the PIN-NH_2_ is still highly efficient and selective, with only minor decreases in the CO_2_ adsorption capacity and selectivity. Moreover, we demonstrated that the CO_2_ adsorption process is fully reversible. The above advantages make the porous organic polymer PIN-NH_2_ a outstanding candidate for CO_2_ separation material, more importantly, the proposed coefficient effect is expected to be a new rationale for the design and fabrication of CO_2_ capture materials for applications in natural gas purification, greenhouse gas reduction, *etc.*

## Conflicts of interest

There are no conflicts to declare.

## Supplementary Material

RA-009-C9RA01532A-s001

## References

[cit1] Gasser T., Kechiar M., Ciais P., Burke E. J., Kleinen T., Zhu D., Huang Y., Ekici A., Obersteiner M. (2018). Nat. Geosci..

[cit2] Cooper A. I. (2015). Nature.

[cit3] Dani A., Crocellà V., Magistris C., Santoro V., Yuana J., Bordiga S. (2017). J. Mater. Chem. A.

[cit4] Naeem M. A., Armutlulu A., Imtiaz Q., Donat F., Schäublin R., Kierzkowska A., Müller C. R. (2018). Nat. Commun..

[cit5] Cavalcanti L. P., Kalantzopoulos G. N., Eckert J., Knudsen K. D., Fossum J. O. (2018). Sci. Rep..

[cit6] Ghalei B., Sakurai K., Kinoshita Y., Wakimoto K., Isfahani A. P., Song Q., Doitomi K., Furukawa S., Hirao H., Kusuda H., Kitagawa S., Sivaniah E. (2017). Nat. Energy.

[cit7] Rochelle G. (2009). Science.

[cit8] Chang G., Wang Y., Wang C., Li Y., Xu Y., Yang Li. (2018). Chem. Commun..

[cit9] Wang J., Zhang P., Liu L., Zhang Y., Yang J., Zeng Z., Deng S. (2018). Chem. Eng. J..

[cit10] Zhang P., Zhong Y., Ding J., Wang J., Xu M., Deng Q., Zeng Z., Deng S. (2019). Chem. Eng. J..

[cit11] Islamoglu T., Kim T., Kahveci Z., El-Kadri O. M., El-Kaderi H. M. (2016). J. Phys. Chem. C.

[cit12] Kim S., Lee Y. M. (2015). Prog. Polym. Sci..

[cit13] Xiang Z., Mercado R., Huck J. M., Wang H., Guo Z., Wang W., Cao D., Haranczyk M., Smit B. (2015). J. Am. Chem. Soc..

[cit14] Chang G., Yang L., Yang J., Stoykovich M. P., Deng X., Cui J., Wang D. (2018). Adv. Mater..

[cit15] Yang P., Yang L., Wang Y., Song L., Yang J., Chang G. (2019). J. Mater. Chem. A.

[cit16] Flaig R. W., Osborn Popp T. M., Fracaroli A. M., Kapustin E. A., Kalmutzki M. J., Altamimi R. M., Fathieh F., Reimer J. A., Yaghi O. M. (2017). J. Am. Chem. Soc..

[cit17] Thakkar H., Eastman S., Al-Mamoori A., Hajari A., Rownaghi A. A., Rezaei F. (2017). ACS Appl. Mater. Interfaces.

[cit18] Alabadi A., Abbood H. A., Li Q., Jing N., Tan B. (2016). Nature.

[cit19] Yang L., Chang G., Wang D. (2017). ACS Appl. Mater. Interfaces.

[cit20] Vishnyakov A., Ravikovitch P. I., Neimark A. V. (1999). Langmuir.

[cit21] Liu J., Thallapally P. K., McGrail B. P., Brown D. R. (2012). Chem. Soc. Rev..

[cit22] Ghanem B. S., Hashem M., Harris D. M., Msayib K. J., Xu M., Budd P. M., Chaukura N., Book D., Tedds S., Walton A., McKeown N. B. (2010). Macromolecules.

[cit23] Ding X. S., Li H., Zhao Y. C., Han B. H. (2015). Polym. Chem..

[cit24] Zhang C., Zhu P. C., Tan L. X., Luo L. N., Liu Y., Liu J. M., Ding S. Y., Tan B. X., Yang L., Xu H. B. (2016). Polymer.

[cit25] Lu J., Zhang J. (2014). J. Mater. Chem. A.

[cit26] Dawson R., Adams D. J., Cooper A. I. (2011). Chem. Sci..

[cit27] Lu W., Yuan D., Sculley J., Zhao D., Krishna R., Zhou H. C. (2011). J. Am. Chem. Soc..

[cit28] Chang G., Shang Z., Tao Y., Yang L. (2016). J. Mater. Chem. A.

[cit29] Chang G., Yang L., Yang J., Huang Y., Cao K., Ma J., Wang D. (2016). Polym. Chem..

[cit30] Balzer C., Cimino R. T., Gor G. Y., Neimark A. V., Reichenauer G. (2016). Langmuir.

[cit31] Kizzie A. C., Wong-Foy A. G., Matzger A. J. (2011). Langmuir.

[cit32] Liu J., Tian J., Thallapally P. K., McGrail B. P. (2012). J. Phys. Chem. C.

