# Supplementary material for: Facile synthesis of aminated indole-based porous organic polymer for highly selective capture of CO2 by the coefficient effect of π–π-stacking and hydrogen bonding
Source: RSC Adv. 2019 Apr 16;9(21):11851–4. doi: 10.1039/c9ra01532a (PMC9063548; doi:10.1039/c9ra01532a)
Supplement: RA-009-C9RA01532A-s001 [file RA-009-C9RA01532A-s001.pdf]

Electronic Supplementary Material (ESI) for RSC Advances.
This journal is © The Royal Society of Chemistry 2019

Supporting Information

Facile Synthesis Aminated Indole-based Porous Organic Polymer for Highly Selective Capture CO_2 by the Coefficient Effect of π-π-stacking and Hydrogen Bonding

Qiang He, Yi Xu, and Xiaoqiang Yang*

School of Aviation Engineering Institute, Civil Aviation Flight University of China, Guanghan , 618307, People's Republic of China.

Corresponding Authors. E-mail: heqiangxy@126.com

Table of contents

1. Main materials..S3

2. Measurements S3

3. Preparation of porous organic polymer material PIN-NH_2 aerogel..........S4

4. Structural characterization of PIN-NH_2 ....S4

5. BET specific surface area plots of PIN-NH_2..S5

6. The XRD of porous organic polymer PIN-NH_2.......................................S6

7. Thermal stability of porous organic polymer PIN-NH_2...........................S7

8. Isosteric heat of CO_2 adsorption for PIN-NH_2...S7

9. Gas adsorption isotherms of PIN-NH_2...S8

10. The comparation the CO_2 capacity, CO_2/N_2, CO_2/CH_4 selectivity S9

11. The dynamic breakthrough separation tests...S10

12. The adsorption measurements..S10

13. Simulation method S12

14. References S13

1. Main materials

4-Aminoindole (97%), 1,2-dichloroethane (AR), iron (III) chloride ($FeCl_3$, AR), formaldehyde dimethyl acetal (AR), methanol (AR), sulfuric acid (H_2SO_4, 98%), anhydrous sodium sulfite (Na_2SO_3, AR), sodium hydroxide (NaOH, AR), hydrochloric acid (HCl, 12 M) and acetone (AR) were purchased from Shanghai Reagent (Shanghai, China). 4-Aminoindole was stored in a refrigerator at 4 °C. Other reagents were of analytical grade and used without any further purification. The rest of the materials and reagents were obtained from different commercial sources and used without further purification.

2. Measurements

FT-IR spectrum was recorded on a Nicolet 6700 FTIR spectrometer. Solid-state cross-polarization magic-angle-spinning (CP/MAS) NMR spectrum was recorded on a Bruker Avance III 400 NMR spectrometer. The elemental analysis characterization technique was performed using a Vario EL III apparatus. Thermogravimetric analysis (TGA) was performed on a Setarma TG-92 at a heating rate of 10 °C/min under nitrogen atmosphere. Scanning electron microscopy (SEM) was recorded on an S-4800 (Hitachi Ltd) field emission scanning electron microscope. Morphological observation was performed with a Tecnai G2 F20 S-TWIN (FEI Company) transmission electron microscope. The nitrogen adsorption and desorption isotherms were measured at 77 K using a Micromeritics AR-JW-BK112 instrument. The sample was treated at 120 °C for 24 h before the measurement. The surface area was calculated by Brunauer-Emmett-Teller (BET)

equation ($0.01 < P/P_0 < 0.1$). The pore-size-distribution (PSD) curve was obtained from the adsorption branch using non-local density functional theory (NL-DFT) method.

3. Preparation of porous organic polymer material PIN-NH_2 aerogel

4-Aminoindole (0.528 g, 4 mmol) was added to anhydrous 1,2-dichloroethane (40 mL) under a flow of N_2, followed by formaldehyde dimethyl acetal (0.4566 g, 6 mmol). $FeCl_3$ (1.30 g, 8 mmol) was added and heated to 80 °C for 18 h (Scheme S1). The solid product was collected by filtration and washed with methanol, HCl (10%) and deionized water to remove the residual $FeCl_3$. The product was further purified by soxhlet extraction in methanol, dried in vacuum at 60 °C for 24 h.

FT-IR spectrum (KBr pellet, cm^{-1}): 3438, 2999, 2927, 1630, 1480, 1388, 1027; Anal. calcd for $(C_9H_7N_2)_n$: C, 75.52; H, 4.89; N, 19.58; found: C,76.29; H, 4.02; N, 19.69.

4. Structural characterization of PIN-NH_2

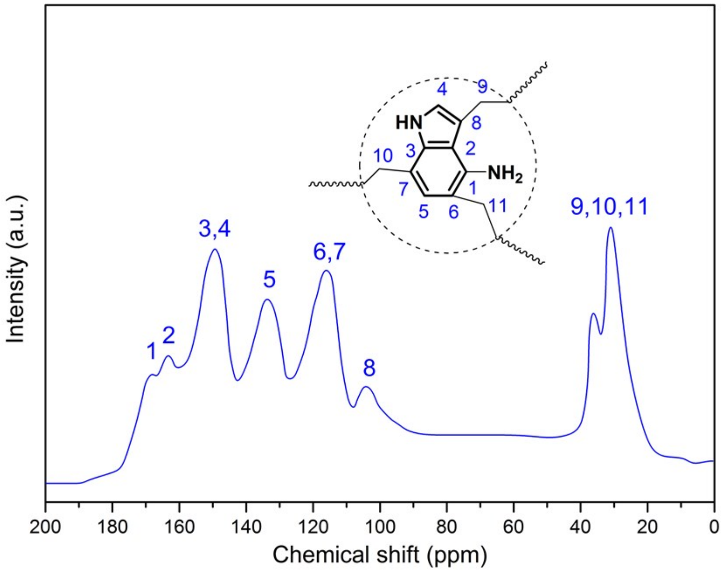

Figure S1. ^{13}C CP/MAS NMR spectrum of PIN-NH_2.

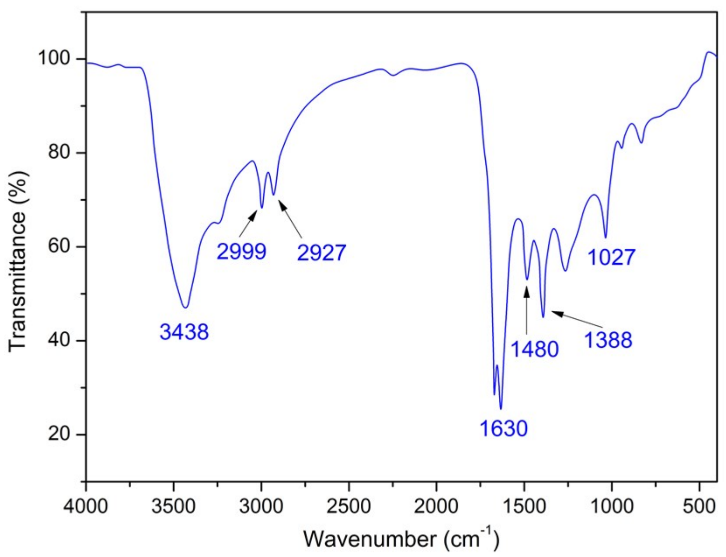

Figure S2. FT-IR spectrum of PIN-NH_2.

5. BET specific surface area plots of PIN-NH_2

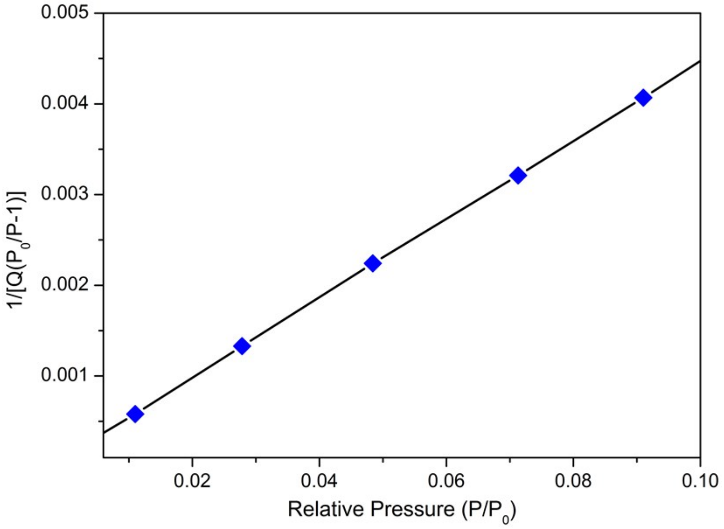

Figure S3. BET specific surface area plots of PIN-NH_2.

6. The XRD of porous organic polymer PIN-NH_2

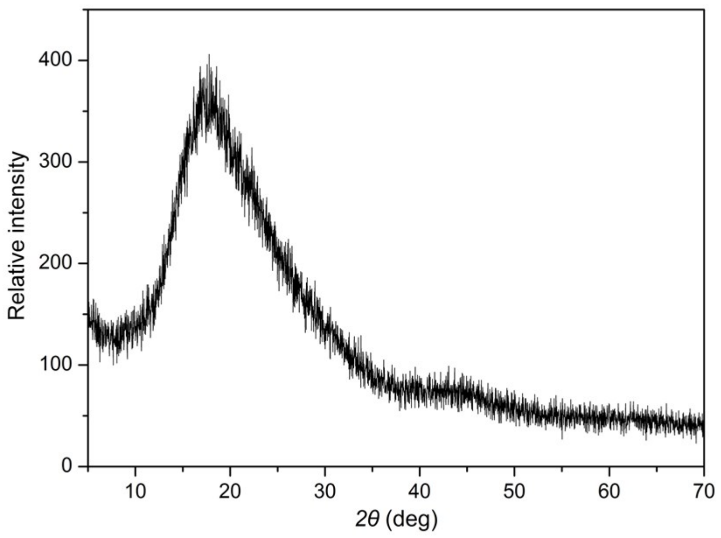

Figure S4. The XRD pattern of porous organic polymer PIN-NH_2.

7. Thermal stability of porous organic polymer PIN-NH_2

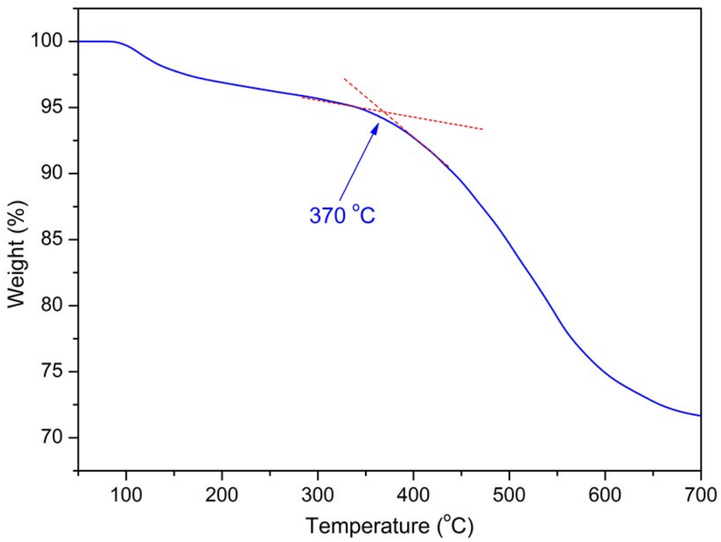

Figure S5. TG curve of PIN-NH_2 in nitrogen.

8. Isosteric heat of CO_2 adsorption for PIN-NH_2

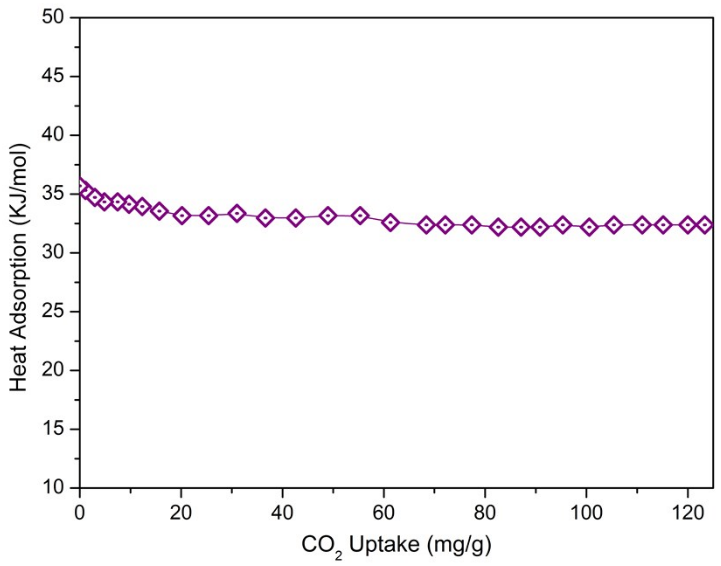

Figure S6. Isosteric heat of CO_2 adsorption for PIN-NH_2.

9. Gas adsorption isotherms of PIN-NH_2

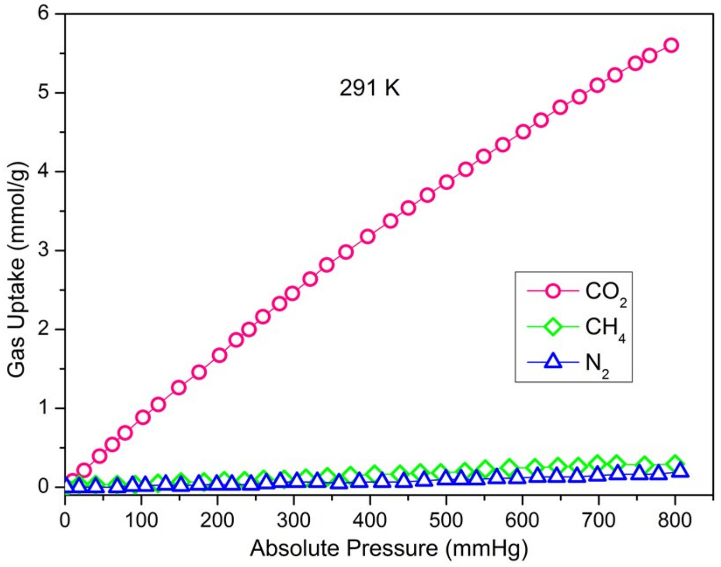

Figure S7. Gas adsorption isotherms of PIN-NH_2 at 291 K.

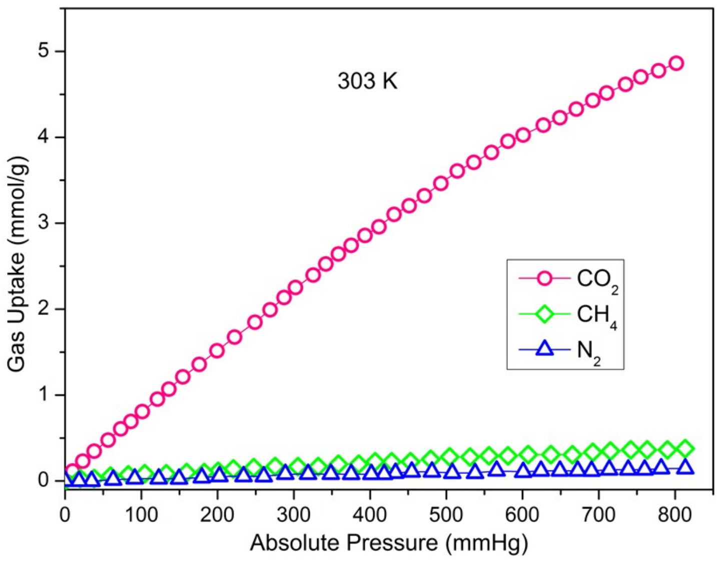

Figure S8. Gas adsorption isotherms of PIN-NH_2 at 303 K.

10. The comparation the CO_2 capacity, CO_2/N_2, CO_2/CH_4 selectivity

Table S1. The comparation the CO_2 capacity, CO_2/N_2, CO_2/CH_4 selectivity.

adsorbents	CO_2 capacity	CO_2/N_2 selectivity	CO_2/CH_4 selectivity	reference
**PIN-NH_2**	27.7 wt% (273 K, 1 bar)	137 (273 K, 1 bar)	34 (273 K, 1 bar)	
**HKUST-1 (CuBTC)**	10.7 mmol/kg (298 K, 35 bar)	-	5 (303 K, 1 bar)	[1]
**MAPOP-1**	12.6 wt% (273 K, 1 bar)	-	4.1 (273 K, 1 bar)	[2]
**MAPOP-2**	12.2 wt% (273 K, 1 bar)	-	4.1 (273 K, 1 bar)	[2]
**MAPOP-3**	11.6 wt% (273 K, 1 bar)	-	4.3 (273 K, 1 bar)	[2]
**MAPOP-4**	13.5 wt% (273 K, 1 bar)	-	4.8 (273 K, 1 bar)	[2]
**PINAA**	5.83 mmol/g (273 K, 1 bar)	83 (273 K, 1 bar)	22 (273 K, 1 bar)	[3]
**PAA**	3.25 mmol/g(273 K, 1 bar)	81 (273 K, 1 bar)	20 (273 K, 1 bar)	[3]
**PIN**	4.92 mmol/g (273 K, 1 bar)	33 (273 K, 1 bar)	17 (273 K, 1 bar)	[3]
**HTP-B**	10.3 wt % (273 K, 1 bar)	16 (273 K, 1 bar)	-	[4]

11. The dynamic breakthrough separation tests

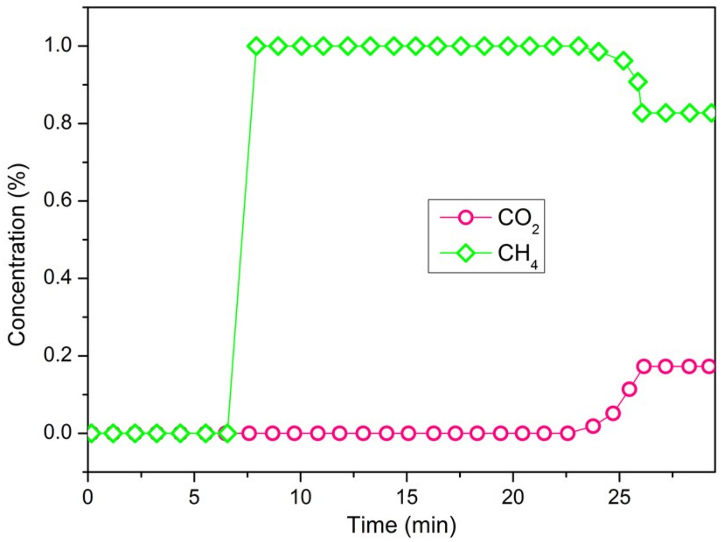

Figure S9. The dynamic breakthrough separation curves for CO_2/CH_4 mixture gas.

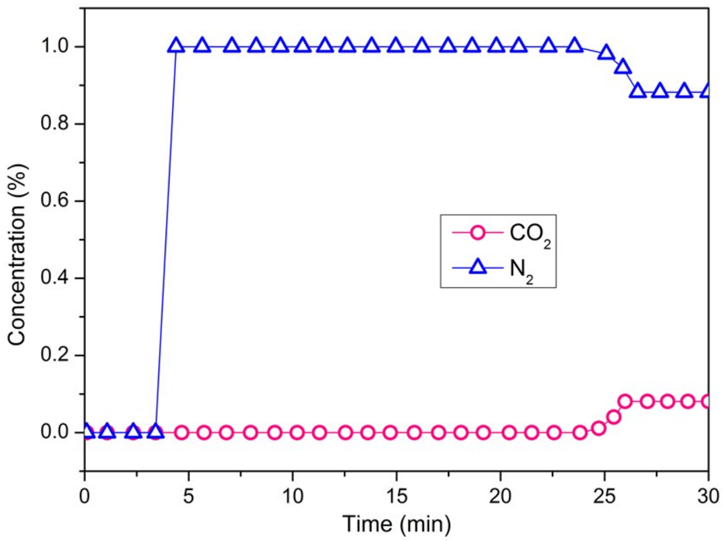

Figure S10. The dynamic breakthrough separation curves for CO_2/N_2 mixture gas.

12. The adsorption measurements

The CO_2, N_2 and CH_4 adsorption measurements were carried out using a Micromeritics AR-JW-BK112 analyzer at 273, 291 and 303 K and up to 1 bar. The temperature during

adsorption and desorption was kept constant using a circulator. Highly pure gases CO_2 (99.999%), N_2 (99.999%) and CH_4 (99.99%) were employed for the measurements. The Clausius-Clapeyron equation was employed to calculate the enthalpies of adsorption for CO_2 on the network. The data was fit using the equation: $(\ln P)_n = -(Q_{st}/R)(1/T)+C$, where P is the pressure, n is the amount adsorbed, T is the temperature, R is the universal gas constant and C is a constant. The isosteric heat of adsorption Q_{st} was subsequently obtained from the slope of plots of $(\ln P)_n$ as a function of $1/T$.[5] The adsorption selectivities have been defined as $S = (x_i/y_i)/(x_j/y_j)$, where x_i and y_i (x_j and y_j) are the molar fractions of component 1 (component 2) in the adsorbed and bulk phases, respectively.[6] In the calculation, the ratio of CO_2/N_2 is 15/85 and the ratio of CO_2/CH_4 is 5/95, which is the typical composition of flue gas and natural gas, respectively. The regeneration experiments were carried out with an Micromeritics AR-JW-BK112 analyzer, the sample was saturated with CO_2 up to 1 bar at 291 K. The recovered adsorbent was evacuated at room temperature for 10 min and then reused for adsorption. Breakthrough curve measurements were performed using a home-made gas-flow system and the experiment is conducted as follows: The adsorbent PIN-NH_2 was filled into the column with a length of 150 mm and an internal diameter of 5 mm. And before the test, the column was activated in flow of Ar for 24 h. The pressure in the column was maintained at 3.1 MPa. The experiments were performed at 298 K. For the CO_2/N_2 mixture gas, the gas fraction was CO_2/N_2=15/85 (v/v) with a space velocity of 5 min^{-1}. For the CO_2/CH_4, the gas component was CO_2/CH_4=5/95 (v/v) with a space velocity of 5 min^{-1}. The flow rates of

all of the pure gases were regulated by mass flow controllers (0-100 mL min^{-1}). The gas mixture was first sent to the gas chromatograph through bypass line and measured its component before the breakthrough measurements. The relative amounts of the effluent gases passing through the column were monitored by GC.

13. Simulation method

To illustrate the molecular mechanism, we used density functional theory (DFT)[7,8] to investigate the interaction of the PIN-NH_2 model compound with CO_2. It was calculated at the M06-2X level with the aug-cc-pVDZ basis set and the resolution-of-identity spin-component-scaling Möller-Plesset second-order perturbation theory (RI-scs-MP2) level with the aug-cc-pVTZ basis set.[9-11] The geometries were fully optimized without symmetry constraints at each calculation level. The M06-2X functional (hybrid-meta GGA with dispersion correction) has shown good performance in the investigation of the dispersion interaction as well as the electrostatic interaction (H-bonding, H-π interaction, π-π interaction, additional electrostatic and induction energies of neutral and charged dimeric systems).[12,13] Single point calculations using the RI-coupled cluster theory with single, double and perturbative triple excitations (RI-CCSD(T)) were performed by employing the aVTZ and aug-cc-pVQZ (aVQZ) basis sets at the RI-scs-MP2/aVTZ geometries. The CO_2-BEs were calculated at the complete basis set (CBS) limit at the RI-CCSD(T) level with the aVTZ and aVQZ basis sets by employing the extrapolation approximation.[14,15] The complete basis set energies were S-19 estimated with the extrapolation scheme utilizing the electron correlation error proportional to N-3 for the

aug-cc-pVNZ basis set (N=3:T, N=4:Q). It is generally known that the zero-point-energy (ZPE)-uncorrected BE(-ΔEe) is closer to the experimental CO_2-adsorption enthalpy (ΔHads) than the ZPE-corrected BE(-ΔE_0).[16,17] Therefore, the values of -ΔEe are reported as the CO_2-BEs.

14. References

[1] J. Liu, P. K. Thallapally, B. P. McGrail, D. R. Brown, J. Chem. Soc. Rev. 2012, 41, 2308.

[2] X. S. Ding, H. Li, Y. C. Zhao and B. H. Han, Polym. Chem. 2015, 6, 5305.

[3] L. Yang, G. Chang and D. Wang, ACS Appl. Mater. Interfaces 2017, 9, 15213.

[4] C. Zhang, P. C. Zhu, L. X. Tan, L. N. Luo, Y. Liu, J. M. Liu, S. Y. Ding, B. X. Tan, L. Yang and H. B. Xu, Polymer, 2016, 82, 100.

[5] Dunne JA, Mariwala R, Rao M, Sircar S, Gorte RJ, Myers AL (1996) Calorimetric heats of adsorption and adsorption isotherms. 1. O_2, N_2, Ar, CO_2, CH_4, C_2H_6, and SF_6 on silicalite. Langmuir 12:5888-5895

[6] Yin Y, Tan P, Liu XQ, Zhu J, Sun LB (2014) Constructing a confined space in silica nanopores: an ideal platform for the formation and dispersion of cuprous sites. J Mater Chem A 2:3399-3406

[7] Altarawneh, S.; Behera, S.; Jena, P.; El-Kaderi, H. M. New Insights into Carbon Dioxide Interactions with Benzimidazole-Linked Polymers. Chem. Commun. 2014, 50, 3571-3574.

[8] Mercado, R.; Vlaisavljevich, B.; Lin, L.; Lee, K.; Lee, Y.; Mason, J. A.; Xiao, D. J.; Gonzalez, M. I.; Kapelewski, M. T.; Neaton J. B.; Smit, B. Force Field Development from Periodic Density Functional Theory Calculations for Gas Separation Applications Using Metal-Organic Frameworks. J. Phys. Chem. C 2016, 120, 12590-12604.

[9] Gerenkamp, M.; Grimme, S. Spin-Component Scaled Second-Order Møller-Plesset Perturbation Theory for the Calculation of Molecular Geometries and Harmonic Vibrational Frequencies. Chem. Phys. Lett., 2004, 392, 229-235. S-20

[10] Zhao, Y.; Truhlar, D. G. Comparative DFT Study of van der Waals Complexes: Rare-Gas Dimers, Alkaline-Earth Dimers, Zinc Dimer, and Zinc-Rare-Gas Dimers. J. Phys. Chem. A, 2006, 110, 5121-5129.

[11] Grimme, S. Improved second-order Møller-Plesset Perturbation Theory by Separate Scaling of Parallel- and Antiparallel-Spin Pair Correlation Energies. J. Chem. Phys., 2003, 118, 9095-9102.

[12] Kolaski, M.; Kumar, A.; Singh, N. J.; Kim, K. S. Differences in Structure, Energy, and Spectrum between Neutral, Protonated, and Deprotonated Phenol Dimers: Comparison of Various Density Functionals with AB Initio Theory. Phys. Chem. Chem. Phys., 2011, 13, 991-1001.

[13] Lee, H. M.; Youn, I. S.; Saleh, M.; Lee, J. W.; Kim, K. S. Interactions of CO2 with Various Functional Molecules. Phys. Chem. Chem. Phys., 2015, 17, 10925-10933.

[14] Min, S. K.; Lee, E. C.; Lee, H. M.; Kim, D. Y.; Kim, D.; Kim, K. S. Complete Basis Set Limit of Ab Initio Binding Energies and Geometrical Parameters for Various Typical Types of Complexes. J. Comput. Chem., 2008, 29, 1208-1221.

[15] Helgaker, T.; Klopper, W.; Koch, H. Noga, J. Basis-Set Convergence of Correlated Calculations on Water. J. Chem. Phys., 1997, 106, 9639-9646.

[16] Saleh, M.; Lee, H. M.; Kemp, K. C.; Kim, K. S. Highly Stable CO2/N2 and CO2/CH4 Selectivity in Hyper-Cross-Linked Heterocyclic Porous Polymers. ACS Appl. Mater. Interfaces 2014, 6, 7325-7333.

[17] Vaidhyanathan, R.; Iremonger, S. S.; Shimizu, G. K. H.; Boyd, P. G.; Alavi, S.; Woo, T. K. Direct Observation and Quantification of CO2 Binding Within an Amine-Functionalized Nanoporous Solid. Science, 2010, 330, 650-653.
